# Predictive value of NLR, TILs (CD4+/CD8+) and PD-L1 expression for prognosis and response to preoperative chemotherapy in gastric cancer

**DOI:** 10.1007/s00262-021-02960-1

**Published:** 2021-05-19

**Authors:** Ina Valeria Zurlo, Mattia Schino, Antonia Strippoli, Maria Alessandra Calegari, Alessandra Cocomazzi, Alessandra Cassano, Carmelo Pozzo, Mariantonietta Di Salvatore, Riccardo Ricci, Carlo Barone, Emilio Bria, Giampaolo Tortora, Luigi Maria Larocca, Michele Basso, Maurizio Martini

**Affiliations:** 1 Fondazione Policlinico Universitario A. Gemelli IRCCS, Largo A. Gemelli 8, 00168 Rome, Italy; 2 Division of Pathology, Department of Health Science and Public Health, Università Cattolica del Sacro Cuore, Largo Francesco Vito 1, 00168 Rome, Italy; 3 Division of Oncology, Department of Medicine and Translational Surgery, Università Cattolica del Sacro Cuore, Largo F. Vito 1, 00168 Rome, Italy

**Keywords:** Gastric cancer, Neoadjuvant chemotherapy, Predictive factors, Precision medicine, Immunological status

## Abstract

The combination of perioperative chemotherapy plus complete surgical resection is currently accounted as the first-choice strategy in patients with locally advanced Gastric Cancer (LAGC). Nevertheless, the partial response rate makes it necessary to search biological parameters useful to select patients who would benefit most from neoadjuvant chemotherapy (NAD-CT). We performed a retrospective analysis on a cohort of 65 LAGC cases, EBV negative and without MMR defect, submitted to perioperative chemotherapy plus surgical resection. We evaluated the neutrophil-lymphocytes ratio (NLR) in peripheral blood, the TILs density (reported as CD4/CD8 tissue ratio) and PD-L1 expression by immunohistochemistry on bioptic tissues before the treatment. Results were correlated with the biological features, histological response (TRG) and clinical outcome (PFS and OS). We found that NLR, TILs and PD-L1 expression showed a significant correlation with TNM stage, lymphovascular invasion and response to NAD-CT (TRG). Correlating the NLR, TILs and PD-L1 expression with PFS and OS, we found that patients with lower NLR levels (< 2.5 ratio), lower TILs (< 0.2 ratio) and higher PD-L1 level (CPS ≥ 1) had a significantly better PFS and OS than those with higher NLR, higher TILs and lower PD-L1 expression (*p* < 0.0001). Multivariate and multiple regression analyses confirmed the predictive and prognostic role of all three parameters, especially when all three parameters are combined. Our study demonstrated that pre-treatment NLR, TILs and PD-L1 expression are predictive and prognostic parameters in NAD-CT-treated LAGC suggesting a pivotal role of the systemic and tumor microenvironment immunological profile in the response to chemotherapy.

**Supplementary Information:**

The online version contains supplementary material available at 10.1007/s00262-021-02960-1.

## Background

Gastric cancer (GC) stands as the fifth most frequently diagnosed malignancy and the third leading cause of cancer death [1]; in Western countries, about two-thirds of GC patients are diagnosed with locally advanced cancer (LAGC) or metastatic disease [2].

The combination of perioperative chemotherapy plus complete surgical resection (R0) is currently considered as the first-choice strategy to improve progression-free survival (PFS) and overall survival (OS) in LAGC patients [3–5].

Nevertheless, while it is true that latest years brought to significant advances both in surgery and combined drug regimens, the overall response rate to chemotherapy is still less than 50%, keeping the prognosis rather dismal [3–5]. Therefore, it would be advantageous to select patients who would benefit most from neoadjuvant chemotherapy (NAD-CT) and who might gain the best survival advantage [6, 7].

In this way, several studies have highlighted that tumor immune infiltrations [7–9], by defining neoplasms either as immunologically “cold,” when they exhibit a low level of tumor infiltrating lymphocytes (TILs), or as immunologically “hot,” when TILs’ level is high, could not only be a prognostic marker in GC in general terms—thus supporting the role of immunotherapy (IT) in GC itself [10, 11]—but could also play a role in predicting GC response to NAD-CT [7, 12, 13]. However, this topic is still debated, since current literature data are conflicting and ultimately leading to disagreement on both type and association of biomarkers to be used to investigate GC-related “immunological status” [6, 7, 12].

Recently, systemic immune-inflammation indexes, based on routinely measurable peripheral blood parameters—such as neutrophil-to-lymphocyte ratio (NLR)—have been proposed as manageable prognosticators and surrogates of cancer-related inflammation in a variety of neoplasms [14], including GC, also after NAD-CT treatment [6, 7, 15].

Furthermore, lymphocyte sub-sets in GC have also been considered, hinting a significant correlation between TILs, expressed as CD4+/CD8+ T cells tissue ratio, and survival [12, 16, 17], while other studies focused on high microsatellite instability, MSI-H and PD-L1 expression, as a potential indicator of resistance to NAD-CT [12, 18, 19].

Based on the latest evidence, we performed a retrospective monocentric analysis, in a cohort of NAD-CT treated locally advanced GCs, EBV negative and with normal expression of mismatch repair (MMR) proteins, to evaluate the baseline NRL, TILs density (reported as CD4+/CD8+ T cells tissue ratio) and PDL-1 expression, as indicators of the GC immunological status, and their correlation with the main clinical and biological features, including response to therapy.

## Methods

### Patients’ features

This exploratory monocentric retrospective analysis was performed at the Unit of Medical Oncology and Unit of Pathology of Fondazione Policlinico Universitario “Agostino Gemelli”—IRCCS, Università Cattolica del Sacro Cuore, Rome. Clinical and pathological records from 112 CT-*naïve* patients with LAGC treated with NAD-CT from January 2012 to January 2017 were reviewed. Inclusion and exclusion criteria, chemotherapy regimen, post-treatment follow-up examinations and other clinical parameters were reported in supplementary material. Finally, 65 patients who met the inclusion criteria were enrolled in this study, while 47 patients were excluded. The main clinical and biological characteristics of the cohort are shown in Table 1.

**Table 1:** Patient characteristics

	*n* = 65
Age, mean (± SD)	63 (9.3)
Gender [*n* (%)]	
Male	41 (63.1)
Female	24 (36.9)
TNM stage* [*n* (%)]	
IIIB	35 (53.85)
IIIC	30 (46.15)
Tumor site [*n* (%)]	
Upper	25 (38.5)
Middle	18 (27.7)
Lower	22 (33.8)
Histological subtype [*n* (%)]	
Intestinal	31 (47.7)
Diffuse	34 (52.3)
HER2 status [*n* (%)]	
HER2+	15 (23.1)
HER2−	50 (76.9)
Response to NAD-CT [*n* (%)]	
TRG 1–2	34 (52.3)
TRG 3–5	31 (47.7)
PFS, mean (months, SD)	26.3 (18.1)
OS, mean (months, SD)	34.6 (18.7)
LVI [*n* (%)]	
Yes	31 (47.7)
No	34 (52.3)
Perineural infiltration (%)	
Yes	20 (30.8)
No	45 (69.2)
NLR pre-chemotherapy (%)	
≥ 2.5	35 (53.8)
< 2.5	30 (46.2)
NLR post-chemotherapy (%)	
≥ 2.5	29 (44.6)
< 2.5	36 (55.4)
CD4+/CD8 + ratio (%)	
≥ 0.2	32 (49.2)
< 0.2	33 (50.8)
PD-L1 expression, CPS (%)	
< 1	34 (52.3)
≥ 1	31 (47.7)

* According to the 8th edition of the American Joint Committee on Cancer (AJCC) guidelines. SD, standard deviation. PFS, progression-free survival. OS, overall survival. LVI, lympho-vascular invasion.

### Blood parameters

Venous blood samples were taken at diagnosis, before NAD-CT, and four weeks or more after the last dose of chemotherapy and within 1 week before the surgical treatment. Hence, baseline or pre-treatment NLR (pre-NRL) and post-treatment NLR (post-NRL) were calculated dividing the absolute neutrophil count (ANC) by the absolute lymphocyte count (ALC; NLR = ANC/ ALC). The cut-off values for white blood cells ANC (> 4000/mm^3^ and ≤ 4000/mm^3^), ALC (> 1500/mm^3^ and ≤ 1500/mm^3^) and NLR (> 2.5 and ≤ 2.5) were defined considering the median values and data from previous studies [6, 7].

### HER2 gene amplification

HER2 amplification was performed using the INFORM HER2/neu Dual ISH DNA Probe Cocktail assay (Ventana Medical Systems, Inc, Tucson, Arizona) as previously described (supplementary material) [20].

### TILs (CD4+/CD8+ T-cells ratio) evaluation and PD-L1 expression

TILs were evaluated as the CD4+/CD8+ T-cells ratio. The expression of CD4 + and CD8 + was assessed by immunohistochemistry (IHC; supplementary material) as previously described with few modifications [8, 9, 21].

PD-L1 expression was evaluated using immunohistochemistry (IHC) and anti-PD-L1 rabbit monoclonal antibody (PD-L1 IHC 22C3 pharmDX; Agilent Technolologies, Carpinteria, CA, USA). Detailed methods are reported in supplementary material. Samples were considered PD-L1 negative if CPS was less than 1 and PD-L1 positive if CPS was 1 or more.

### Statistical analysis

The objective of this analysis was to explore the correlation between clinical and biological parameter, TILs density (reported as CD4/CD8 tissue ratio), PDL-1 expression and NLR, in a cohort of patients with LAGC treated with NAD-CT.

Statistical analysis was performed using GraphPad-Prism 5 software (Graph Pad Software, San Diego, CA) and MedCalc version 10.2.0.0 (MedCalc Software, Mariakerke, Belgium; supplementary material) [20, 22].

The evaluation of the tumor response to neoadjuvant treatment was performed using Mandard’s classification system (Tumor Regression Grade, TRG) [23]. Responders were defined as TRG 1–2, and non-responders were defined as TRG 3–5 [24]. Progression Free Survival (PFS) and Overall Survival (OS) were the survival endpoints. PFS was calculated as the time from NAD-CT beginning to any evidence of disease progression (either local/regional relapses or distant metastases) or death, whichever occurred first. OS was calculated as the time from NAD-CT beginning to the patient’s death, due to any cause.

## Results

### Patient characteristics and treatment response

Main clinicopathologic characteristics of our cohort (65 consecutive patients with LAGC treated with NAD-CT) are reported in Table 1.

Mean age at the time of diagnosis was 63 years, and 63% of patients were male. Clinical TNM stage was IIIB in 35 cases (53.85%) and stage IIIC in 30 cases (46.15%), respectively. Twenty-five patients out of 65 (38.5%) had an upper site located GC (Siewert type 2 or 3), 18 out of 65 (27.7%) had a middle site located GC, and 22 out of 65 (33.8%) a lower site GC. Histologically, 31 out of 65 (47.7%) had an intestinal subtype GC, while 34 out of 65 (52.3%) a diffuse subtype GC. HER2 amplification was found in 15 out of 65 cases (23.1%), while 50 patients (76.9%) were negative. The post- NAD-CT response was evaluated according to Mandard-TRG pathological response system: 34 out of 65 patients (55.4%) had a good tumor regression (TRG 1–2), while 29 out of 65 (44.6%) had a poor tumor regression (TRG 3–5).

### Blood neutrophil–lymphocyte ratio

**Fig. 1:**
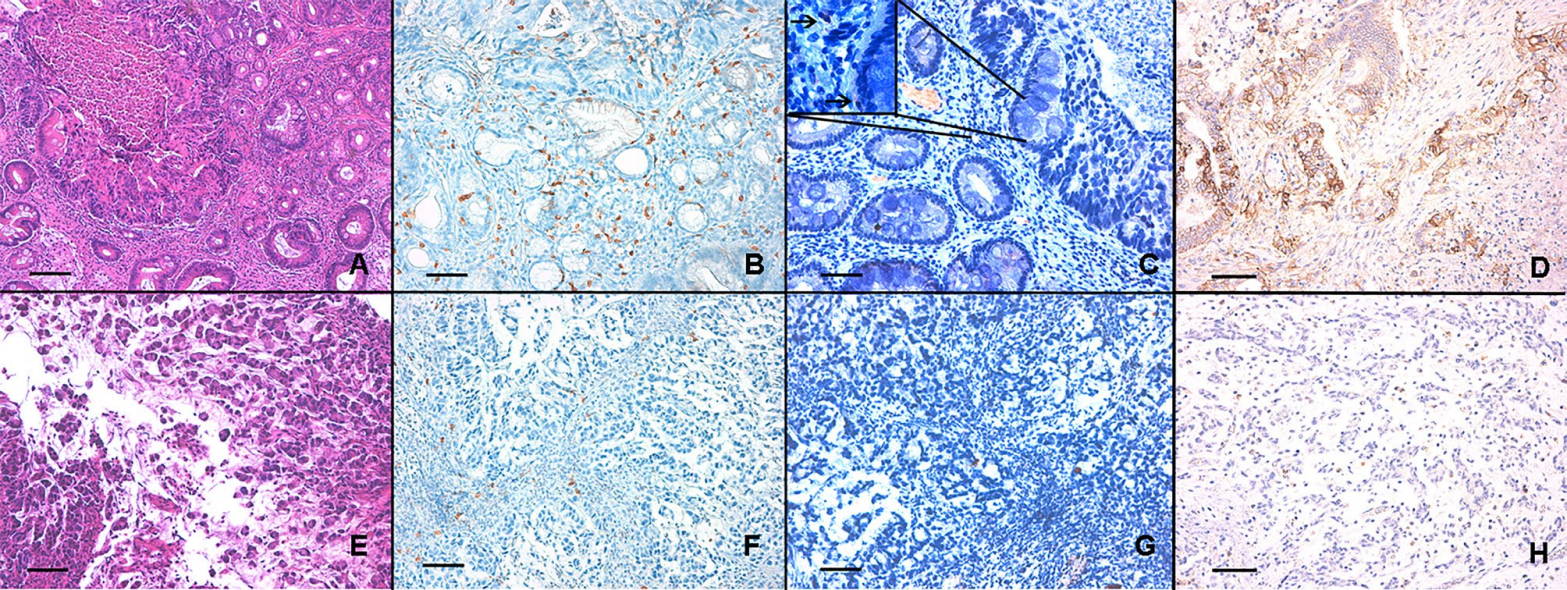
The figure shows two LAGC samples of intestinal and diffuse histotype cancer (panel A and E, respectively, E&E, ×200 magnification, bare scale 150 mm); the CD4 + and CD8 + cell counts (figure B and F for CD8 + cells; figure C and G for CD4 + cells; ×200 magnification, bare scale 150 mm; the box in the panel C shows a detail of the image at  ×400 magnification, where the arrows indicate the CD4 + cells); the PD-L1 expression, evaluated as CPS score (figure D, CPS ≥ 1 and H, CPS < 1; × 200 magnification, bare scale 150 mm)

Mean pre-chemotherapy neutrophil and lymphocyte counts were 4940 and 1801 per mm^3^, respectively. Mean pre-chemotherapy NLR was 3.5 (range 0.57–3.8); by considering 2.5 as the cut-off value for NLR, as reported in the literature data [6], we found that 30 out of 65 patients (46.2%) had a low NLR ratio, while 35 patients (53.8%) had a high NLR ratio (Table 1). On the contrary, mean post-chemotherapy neutrophil and lymphocyte counts were 4010 and 1460 per mm^3^, respectively. Mean post-chemotherapy NLR was 2.74 (range 0.72–12.6); by considering 2.5 as the cut-off value for NLR, we found that 36 out of 65 patients (55.4%) had a low NLR ratio, while 29 patients (44.6%) had a high NLR ratio (Table 1). Correlating the ANC and ALC, in the pre-treatment blood samples, with the clinical and biological parameters of our cohort, we found that male gender was significantly correlated with the ANC (*p* < 0.001; Table 1S), while all other parameters did not have any significant association, either with ANC or with ALC (Table 1S). Moreover, NLR in pre-treatment blood samples showed a significant correlation with TNM stage (*p* = 0.013), lymphovascular invasion (LVI, *p* < 0.001) and TRG (*p* = 0.001; Table 2). Twenty-four patients with a low baseline NLR level remained in this group after first-line chemotherapy (Table 3). By contrast, 6 patients from this group were transferred to the high NLR level group after NAD-CT. Twenty-three patients with a high baseline NLR level retained this level after first-line chemotherapy. By contrast, 12 patients with a high baseline NLR level were transferred to the low NLR level group after NAD-CT. When we correlated the changing in the NLR level with the tumor response to neoadjuvant treatment (TRG), we found that patients who remained in or were transferred to the low NLR level subgroup following NAD-CT exhibited improved response, compared to patients who remained in or were transferred to the high NLR level group (Table 3). Interestingly, we noted a significant decrement in the NRL values between pre- and post-chemotherapy (*p* = 0.0033, paired *t*-test; Fig. 1S, panel A).

**Table 2:** Correlation between clinical and biological parameters with pretreatment NLR, CD4+/CD8 + T-cell tissue ratio and PD-L1 expression

	Pre-treatment NLR				CD4+/CD8+ T-cell tissue ratio				PD-L1, CPS			
	≥ 2.5 (*n*; %)	< 2.5 (*n*; %)	*p*	OR (95% CI)	≥ 0.2 (*n*; %)	< 0.2 (*n*; %)	*p*	OR (95% CI)	CPS ≥ 1 (*n*; %)	CPS < 1 (*n*; %)	*p*	OR (95% CI)
*Age*												
≥ 63	21; 53.85	18; 46.15	1.00	1.00 (0.37; 2.71)	19; 48.72	20; 51.28	1.00	0.97 (0.35; 2.56)	13; 50.00	13; 50.00	0.80	1.17 (0.43; 3.15)
< 63	14; 53.85	12; 46.15			13; 50.00	13; 50.00			18; 46.15	21; 53.85		
*Gender*												
Female	11; 45.83	13; 54.17	0.44	0.60 (0.22; 1.65)	10; 41.67	14; 58.33	0.44	0.78 (0.22; 1.71)	14; 58.33	10; 41.67	0.21	1.98 (0.71; 5.49)
Male	24; 58.54	17; 41.46			22; 53.66	19; 46.34			17; 41.46	24; 58,54		
*TNM stage*												
IIIB	24; 68.57	11; 31.43	**0.013**	3.77 (1.34; 10.56)	22; 62.86	13; 37.14	**0.025**	3.38 (1.22; 9.42)	20; 66.66	10; 33.34	**0.006**	4.36 (1.54; 12.37)
IIIC	11; 36.67	19; 63.33			10; 33.33	20; 66.67			11; 31.42	24; 68.58		
*Tumor site*												
Upper	12; 48.00	13; 52.00	1.00	0.83 (0.35; 1.97)	11; 44.00	14; 56.00	0.60	0.84 (0.35; 2.03)	13; 52.00	12; 48.00	0.49	1.15 (0.48; 2.76)
Middle	9; 50.00	9; 50.00			7; 38.89	11; 61.11			10; 55.55	8; 44.45		
Lower	14; 63.64	8; 36.36			14; 63.64	8; 36.36			8; 36.36	14; 63.64		
*Histotype*												
Intestinal	17; 54.84	14; 45.16	1.00	1.08 (0.41; 2.87)	15; 48.39	16; 51.61	1.00	0.94 (0.35; 2.48)	14; 45.16	17, 54.84	0.80	0.82 (0.31; 2.19)
Diffuse	18; 52.94	16; 47.06			17; 50.00	17; 50.00			17; 50.00	17; 50.00		
*HER2 status*												
HER2 +	11; 73.33	4; 26.67	0.14	2.98 (0.83; 10.63)	9; 60.00	6; 40.00	0.39	1.76 (0.54; 5.69)	4; 26.67	11; 73.33	0.08	0.31 (0.09; 1.11)
HER2 −	24; 48.00	26; 52			23; 46.00	27; 54.00			27; 54.00	23; 46.00		
*LVI*												
Yes	25; 80.65	6; 19.35	** < 0.001**	10 (3.14; 31.81)	23; 74.19	8; 25.81	** < 0.001**	7.98 (2.64; 24.19)	7; 22.58	24; 77.42	** < 0.001**	0.12 (0.04; 0.37)
No	10; 29.41	24; 70.59			9; 26.47	25; 73.53			24; 77.42	10; 22.58		
*PNI*												
Yes	14; 70.00	6; 30.00	0.11	2.67 (0.87; 8.18)	19; 42.22	26; 57.78	0.11	0.39 (0.13; 1.74)	7; 35.00	13; 65.00	0.19	0.47 (0.16; 1.40)
No	21; 46.67	24; 53.33			13; 65	7; 35			24; 5.33	21; 94.67		
*TRG*												
TRG 3–5	21; 67.74	10; 32.26	**0.001**	5.83 (1.99; 17.03)	22; 70.97	9; 29.03	**0.003**	5.11 (1.77; 14.72)	9; 29.03	22; 70.97	** < 0.001**	0.15 (0.05; 0.44)
TRG 1–2	9; 26.47	25; 73.53			11; 32.35	23; 67.65			25; 73.53	9; 26.47		
*PFS, months*												
≥ 26.3	4; 16.67	20; 83.33	** < 0.001**	0.06 (0.18; 0.23)	9; 35	27; 75	** < 0.001**	0.09 (0.03; 0.28)	17; 85.00	3; 15.00	** < 0.001**	12.55 (3.15; 49.9)
< 26.3	31; 75.61	10; 24.39			23; 79.31	6; 20.69			14; 31.11	31; 68.89		
*OS, months*												
≥ 34.6	4; 17.39	19; 82.61	** < 0.001**	0.07 (0.21; 0.27)	1; 4.35	22; 95.65	** < 0.001**	0.02 (0.001; 0.13)	20; 80.00	5; 20.00	** < 0.001**	10.55 (3.17; 35.0)
< 34.6	31; 73.81	11; 26.19			31; 73.81	11; 26.19			11; 27.50	29; 72.50		

*LVI* lymphovascular invasion, *PNI* perineural infiltration

Bold indicates the only significant parameters

**Table 3:** Relationship between changes in the NLR level and tumor response to neoadjuvant treatment (TRG)

Pre-chemotherapy	Post-chemotherapy	TRG 1–2 (*n* = 34)	TRG 3–5 (*n* = 31)	OR	*p* value
NLR < 2.5 (*n* = 30)	NLR < 2.5 (*n* = 24)	15	9	21.21	0.017
	NLR ≥ 2.5 (*n* = 6)	0	6		
NLR ≥ 2.5 (*n* = 35)	NLR < 2.5 (*n* = 12)	11	1	20.63	0.002
	NLR ≥ 2.5 (*n* = 23)	8	15		

### Immunohistochemistry for TILs (CD4+/CD8+ T cells ratio) in GC tissue

**Fig. 2:**
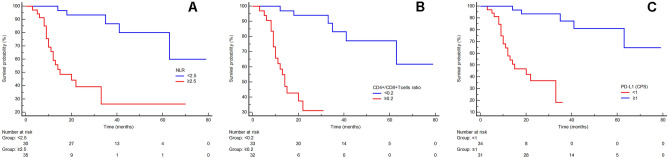
Kaplan–Meier curves for PFS of NAD-CT-treated LAGC patients stratified by pre-treatment NLR (panel A), CD4+/CD8+ T cells tissue ratio (TILs; panel B) and PD-L1 expression (evaluated as CPS score; panel C). Patients with lower NLR < 2.5, TILs < 0.2 and with PD-L1 CPS ≥ 1 (blue-line) were significantly associated with a better PFS (*p* < 0.0001) respect to those patients with NLR ≥ 2.5, TILs ≥ 0.2 and with PD-L1 CPS < 1 (red-line)

Mean pre-chemotherapy CD4+/CD8+ T cells tissue ratio (TILs) was 0.2 (range from 0.03 to 5.53, Fig. 1). Using the TILs’ mean value as the cut-off value, we found that 33 out of 65 patients (50.9%) had a low TILs, while 32 patients (49.2%) had a high TILs (Table 1). Correlating TILs with the clinical and biological parameters of our cohort, we found that TNM stage (*p* = 0.025), LVI (*p* < 0.001) and TRG (*p* = 0.003) were significantly correlated with TILs (Table 2), while none of the other parameters showed any significant association (Table 2). Interestingly, we found that TILs had a direct and significant correlation with pre-treatment NLR value (Spearman *r* = 0.6338; *p* < 0.0001; Fig. 2S, panel A).

### Evaluation of PD‑L1 expression (CPS)

We found that 34 out of 65 patients (52.3%) had a low PD-L1 expression (CPS < 1), while 31 patients (47.7%) had a high PD-L1 level (CPS ≥ 1; Table 1 and Fig. 1). Correlating the PD-L1 expression with the clinical and biological parameters of our cohort, we found that LVI (*p* < 0.001; Table 2), stage (IIIC vs IIIB; *p* = 0.006; Table 2) and TRG (*p* < 0.001; Table 2) were significantly correlated with PD-L1 level, while none of other parameters showed any significant association (Table 2). We also found that PD-L1 expression had an indirect and significant correlation with both pre-treatment NLR value (Spearman *r* = − 0.781; *p* < 0.0001; Fig. 2S, panel B) and TILs (Spearman *r* = − 0.567; *p* < 0.0001; Fig. 2S, panel C).

### Prognostic variables for PFS and OS

**Fig. 3:**
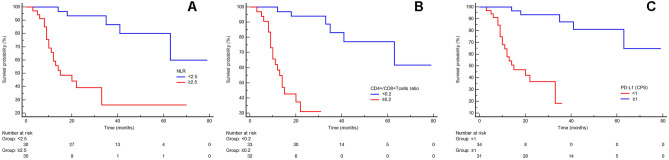
Kaplan–Meier curves for OS of NAD-CT-treated LAGC patients stratified by pre-treatment NLR (panel A), CD4+/CD8+ T cells tissue ratio (TILs; panel B) and PD-L1 expression (evaluated as CPS score; panel C). Patients with lower NLR < 2.5, TILs < 0.2 and with PD-L1 CPS ≥ 1 (blue-line) were significantly associated with a better OS (p < 0.0001) respect to those patients with NLR ≥ 2.5, TILs ≥ 0.2 and with PD-L1 CPS < 1 (red-line)

Mean PFS and mean OS were 26.3 months and 34.6 months, respectively. When we correlate the pre-treatment NLR with PFS and OS, we found that patients with lower NLR levels had a significantly better PFS and OS than those with higher NLR levels (Fig. 2 panel A for PFS: median PFS for lower pre-treatment NLR level 66 months versus median PFS for higher pretreatment NLR level 29 months, *p* < 0.0001, HR 7.21, 95% CI from 3.03 to 17.12; Fig. 3 panel A for OS: median OS for lower pre-treatment NLR level 73 months versus median OS for higher pre-treatment NLR level 37 months, *p* < 0.0001, HR 6.60, 95% CI from 2.86 to 15.23). A similar but lower significant result was observed correlating the post-treatment NLR with PFS and OS (Fig. 1S panel B for PFS: median PFS for lower post-treatment NLR level 61 months versus median PFS for higher post-treatment NLR level 27 months, *p* = 0.001, HR 4.20, 95% CI from 1.78 to 9.91; Fig. 1S panel C for OS: median OS for lower post-treatment NLR level 64 months versus median OS for higher post-treatment NLR level 37 months, *p* = 0.015, HR 2.79, 95% CI from 1.22 to 6.42).

Patients with lower TILs had a significant association with better PFS and OS than those with higher CD4+/CD8+ T-cell tissue ratio (Fig. 2 panel B for PFS: median PFS for patients with lower CD4+/CD8+ T cells ratio 65 months versus median PFS for patients with higher CD4+/CD8+ T cell ratio patients 18 months, *p* < 0.0001, HR 11.88, 95% CI from 4.65 to 30.33; Fig. 3 panel B for OS: median OS for patients with lower CD4+/CD8+ T cell ratio 75 months versus median OS for patients with higher CD4+/CD8 + T cell ratio 27 months, *p* < 0.0001, HR 11.57, 95%CI from 4.76 to 28.12).

Moreover, higher PD-L1 level (CPS ≥ 1) in pretreated GC tissue was significantly associated with a better PFS and OS in comparison with those with lower PD-L1 score (CPS < 1; Fig. 2 panel C for PFS: median PFS for PD-L1 CPS ≥ 1 patients 20 months *versus* median PFS for PD-L1 CPS < 1 patients 67 months, *p* < 0.0001, HR 0.09924, 95%CI from 0.04029 to 0.2445; Fig. 3 panel C for OS: median OS for PD-L1 CPS ≥ 1 patients 28 months versus median OS for PD-L1 CPS < 1 patients 74 months, *p* < 0.0001, HR 0.1154, 95%CI from 0.04865 to 0.2737).

In addition, Kaplan–Meir analysis showed that variables predicting improved PFS and OS were TNM-IIIB (Table 2S, PFS, *p* = 0.0379; OS, *p* = 0.0174), absence of LVI (Table 2S, PFS, *p* = 0.0515; OS, *p* = 0.0196) and TRG 1–2 (Table 2S, PFS, *p* = 0.0244; OS, *p* = 0.0112). No correlation was found with gender, age, tumor site, histological subtypes, HER2 expression nor perineural infiltration (Table 2S).

Multivariate analysis of PFS including pre-treatment NLR, TILs and PD-L1 expression, stage, histological subtypes, TRG and lymphovascular invasion showed that NLR, TILs, PD-L1 expression before NAD-CT were significant predictors (Table 4; *p* = 0.0001 for TILs; *p* = 0.0013 for PD-L1 expression; *p* = 0.0036 for pretreatment NLR). Similarly, multivariate analysis of OS, including pre-treatment NLR, TILs and PD-L1 expression, stage, histological subtypes, TRG and lymphovascular invasion, showed that the independent prognostic variables were pretreatment NLR, TILs and PD-L1 expression (Table 4; *p* = 0.0004 for both TILs and PD-L1 expression; *p* = 0.0012 for pretreatment NLR).

**Table 4:** Multivariate analysis for PFS and OS

	*b*	SE	Wald	*p*	Exp(*b*)	95% CI of Exp(*b*)
*Covariate for PFS*						
CD4+/CD8+ ratio	2.7528	0.7025	15.3540	0.0001	15.6868	3.9584–62.1645
PD-L1 (CPS)	2.1720	0.6765	10.3092	0.0013	0.1139	0.0303–0.4291
Pre-treatment NLR	1.8501	0.6357	8.4692	0.0036	6.3605	1.8295–22.1123
*Covariate for OS*						
CD4+/CD8+ ratio	2.8712	0.8072	12.6524	0.0004	17.6577	3.6295–85.9058
PD-L1 (CPS)	2.5776	0.7249	12.6431	0.0004	0.0760	0.0183–0.3145
Pre-treatment NLR	2.0307	0.6266	10.5042	0.0012	7.6195	2.2314–26.0178

*b* = coefficient estimates; SE = standard error for coefficient estimates *b*; Exp(*b*) = Hazard Ratio value; 95%CI of Exp(*b*) = 95% confidence interval of Hazard Ratio

The predictive value for PFS and OS of each parameter (pre-treatment NLR, PD-L1 expression and TILs) was evaluated, first individually, then combined (two or three parameters), performing a multiple regression analysis. The predictive value was significantly higher only when the three parameters were considered jointly combined (*p* < 0.0001 for both PFS and OS; r partial 0.7329 for PFS and r partial 0. 6157 for OS).

## Discussion

This monocentric study retrospectively investigated the relationship between systemic and tumor microenvironment (TME) immunological profile in patients with LAGC before receiving NAD-CT and their clinicopathologic outcome to identify some parameters which could help in selecting patients who might respond to CT.

According to recent evidence [6, 7, 15], we demonstrated low NLR in the peripheral blood of pre-treated NAD-CT LAGC was significantly associated with a favorable PFS and OS (both *p* < 0.0001), also finding a significant association between low NLR and TRG (*p* = 0.001) and TNM stage (*p* = 0.013) and LVI (*p* ≤ 0.001). At the same time, we evaluated the peritumoral microenvironment, where the immune host cells (mainly lymphocytes) strictly interacted with neoplasm, demonstrating, in agreement with other authors [12, 16, 17], the significant association with low CD4+/CD8+ T cell ratio with PFS, OS (both *p* < 0.001), and the pathological response to NAD-CT (as TRG) in our cohort (*p* = 0.003). Similarly, investigating the expression of PD-L1, we also found, for the first time, that higher level of PD-L1 is associated with better PFS and OS (both *p* < 0.001) and response to neoadjuvant therapy in LAGC (*p* < 0.001). In addition, higher level of CPS (CPS ≥ 1) also had significant association with TNM stage (*p* = 0.006), TRG (*p* < 0.001) and LVI (*p* ≤ 0.001). Lastly, we found that post-treatment NLR levels were consistent with chemotherapeutic efficacy and clinical outcome, suggesting NLR levels following treatment, though less significantly than pre-treatment NRL, may also provide valuable prognostic and predictive information.

Several studies highlighted that the tumor immune status plays a role also in response to neoadjuvant radio-chemotherapy treatments in several human cancers [6–8, 12, 17, 18, 25, 26]. Accordingly, considering the need to identify and select those patients who would benefit most from a neoadjuvant treatment, recent studies have tried to use different immune parameters as indicators of response to neoadjuvant treatments, sometimes finding inconsistent results, especially regarding the role of T cells subtypes [8–12, 17, 18, 27]. This probably depends on different factors such as the inhomogeneity of the analyzed cohorts, different neoadjuvant scheme treatments, as well as the use of different and single parameter to investigate the immune status which is inherently complex and variegated, and whose function is scarcely reducible to only one specific parameter.

Investigating three immunological prechemotherapy parameters, we found that the latter, besides being significantly correlated with better PFS and OS (both *p* < 0.005) in univariate and multivariate analysis, had likewise a direct and significant correlation with each other. In fact, NLR, an expression of the immunosurveillance capacity of the host, the presence of higher levels of CD8 + in the tumor microenvironment, as an index of a patient’s better immune-response, and PD-L1 expression, indicating the tumor’s intrinsic immune-escape capability and consequently also the TME CD8 + T cells level, source of cytokines such as interferon gamma that induced the expression of PD-L1 [28, 29], mirrored some crucial aspects of the tumor/immunity interaction which, if considered together, better select LAGC patients who will benefit most from NAD-CT treatment. In fact, we demonstrated that joining the aforementioned parameters with one another makes stronger correlation between pre-chemotherapy immune status and clinical outcomes (*p* < 0.0001).

Although the chemotherapy tends to deeply modify the host immunity, often with detrimental and myelosuppressive effects, accumulating evidence indicates that the efficacy of conventional anticancer agents does not only involve direct cytostatic/cytotoxic effects, but also relies on the (re)activation of tumor-targeting immune responses, similarly to the abscopal effect due to the radiotherapy [30]. This chemotherapeutic effect might act in synergy with a most reactive, less depressive immune system and higher TME CD8 + [29], whose pre-chemotherapy status could be assessed with NLR, TILs and PD-L1 expression.

By ruling out both altered-MMRP carriers and EBV positive tumors, we managed to minimize confounding effects of other tumor variables on the outcomes and fluorouracil-based NAD-CT [18, 31]. Interestingly, PD-L1 expression, which was typically associated with MSI-H status and maybe indirectly to the fluorouracil-based NAD-CT outcome, as demonstrated in several tumors, showed here a significant correlation with PFS, OS and response to therapy only in LAGC patients displayed higher levels of this immune-suppressing protein. Probably, the upregulation of negative immune checkpoint proteins in gastric tumors is due to the tumor infiltration of effector T cells (especially the CD8 + cells), defined as “T cell inflamed phenotype,” that in turn determine the upregulation of immune checkpoints, and not to a genomic instability [29]. This way, the PD-L1 target in gastric cancer could only be clinically effective (also in NAD-CT) for the subgroup of tumors that contain tumor-infiltrating immune cells and could explain the controversial results in the predictive effects of PD-L1 in response to PD-1/PD-L1 antibodies in GC [32].

Although over the last year, the standard NAD-CT in LAGC changed, after the publication of the FLOT4-trial data [33], we hypothesized that pre-treatment NLR, TILs and PD-L1 expression, even if requires demonstrating, could be a predictive and prognostic parameter also in this new fluorouracil-based regimen.

Unlike Wang et al. [34] who described a significant association between lower PD-L1 expression and HER2 gene amplification in GC, we did not find any significant association between this molecular feature and pre-treatment NLR, TILs and PD-L1 expression, suggesting that this alteration did not have a predictive role in NAD-CT-treated LAGC.

In addition, our data have shown that both the NRL levels before and after NAD-CT have a predictive and prognostic value in patients with LAGC. However, the post-treatment NRL shows less significance than the pre-treatment NRL. This could be partly explained by the bone morrow suppression of the NAD-CT and other biological parameters, as already reported by several authors [9, 35, 36].

Heterogeneity in the immunohistochemical assessment for PD-L1 and TILs (CD4 and CD8) expression both within and between tumor sites is a well-documented phenomenon that could have important implications, especially for PD-L1 accuracy as a predictive biomarker [37–39]. In addition, other factors, such as the use of different antibodies, of different cut-off, of different immunohistochemical platforms and the inter-observer variability could play a role in the evaluation of these two parameters [37, 38]. Notwithstanding, the immunohistochemical assay for PD-L1 and TILs remains today one of the most used markers, especially for immunotherapy, and several studies have shown that it is possible to reduce the impact of the heterogeneity of expression in the evaluation of these two parameters. In this way, we perform the PD-L1 and TILs expression on baseline multisite sampling tumor tissue biopsies (median 4.02, with at least 50% tumor tissue), using the same antibodies and immunohistochemical platform, following standardized and well-defined conditions, and calculating the agreement indices (Cohen’s K index) between the two pathologists (M.M. and R.R., who also went through a formal training program to evaluate CPS by the 22C3 pharmDx assay) in the evaluation of TILs and PD-L1 expression (that were very good: *k* = 0.82 and *k* = 0.87, respectively).

Finally, although the evaluation of NRL, TIL and PD-L1 parameters can be an easily executable analysis and several studies have shown that these parameters, even if individually, could be useful in the selection of LAGC patients for NAD-CT; however, it appears necessary to better understand the molecular mechanisms underlying this association, before these being adopting in clinical practice. To this end, in-vitro models such as co-cultures of patient' immune cells and GC organoid could represent a valid model, in which different types of neoadjuvant treatment could be tested, also in combination with immunotherapy [40].

The main limitations of our study are the retrospective monocentric design and the relatively small though homogeneous-treated cohort of patients, so that our data need to be confirmed in other independent studies including NAD-CT-treated LAGC patients, and our immune parameters should be validated as a prognostic tool.

## Conclusions

In summary, we propose that pre-treatment NLR, TILs and PD-L1 expression may be predictive and prognostic parameters in NAD-CT-treated LAGC suggesting a pivotal role of the tumor inflammatory microenvironment in the response to chemotherapy. These three parameters, as indicators of the patient tumor/immune system status, significantly correlated each other and could help the clinicians to recognize patients who might benefit from a NAD-CT with LAGC.

## Supplementary Information

Below is the link to the electronic supplementary material.

Supplementary file1 (DOCX 21 kb)

Supplementary file2 (TIF 4319 kb)

Supplementary file3 (TIF 2523 kb)

Supplementary file4 (XLSX 12 kb)

Supplementary file5 (DOCX 18 kb)

## Data Availability

The datasets used and/or analyzed during the current study are available from the corresponding author on reasonable request.

## Abbreviations

ALC: Absolute lymphocytes count
ANC: Absolute neutrophil count
CPS: Combined positive score
EBV: Epstein–Barr virus
GC: Gastric cancer
IHC: Immunohistochemistry
LACG: Locally advanced gastric cancer
MSI: Microsatellite instability
MMR: Mismatch repair
NAD-CT: Neoadjuvant chemotherapy
NLR: Neutrophil-lymphocytes ratio
OS: Overall survival
PFS: Progression free survival
TIL: Tumor-infiltrating lymphocytes
TME: Tumor microenvironment
TRG: Tumor regression grade

## References

[1] Bray F, Ferlay J, Soerjomataram I, Siegel RL, Torre LA, Jemal A (2018). Global cancer statistics 2018: GLOBOCAN estimates of incidence and mortality worldwide for 36 cancers in 185 countries. CA A Cancer J Clin.

[2] Orditura M, Galizia G, Sforza V, Gambardella V, Fabozzi A, Laterza MM (2014). Treatment of gastric cancer. World J Gastroenterol.

[3] Bang YJ, Kim YW, Yang HK, Chung HC, Park YK, Lee KH (2012). Adjuvant capecitabine and oxaliplatin for gastric cancer after D2 gastrectomy (classic): a phase 3 open-label, randomised controlled trial. Lancet.

[4] Fuse N, Bando H, Chin K, Ito S, Yoshikawa T, Tsuburaya A (2017). Adjuvant capecitabine plus oxaliplatin after D2 gastrectomy in Japanese patients with gastric cancer: a phase II study. Gastric Cancer.

[5] Fuentes E, Ahmad R, Hong TS, Clark JW, Kwak EL, Rattner DW (2016). Adjuvant therapy completion rates in patients with gastric cancer undergoing perioperative chemotherapy versus a surgery-first approach. J Gastrointest Surg.

[6] Jin H, Zhang G, Liu X, Liu X, Chen C, Yu H (2013). Blood neutrophil-lymphocyte ratio predicts survival for stages III–IV gastric cancer treated with neoadjuvant chemotherapy. World J Surg Oncol.

[7] He Q, Li G, Ji X, Ma L, Wang X, Li Y (2017). Impact of the immune cell population in peripheral blood on response and survival in patients receiving neoadjuvant chemotherapy for advanced gastric cancer. Tumor Biol.

[8] Fridman WH, Galon J, Dieu-Nosjean M, Cremer I, Fisson S, Damotte D, Dranoff G (2010). Immune infiltration in human cancer: prognostic significance and disease control. Cancer immunology and immunotherapy, current topics in microbiology and immunology.

[9] Kim JW, Nam KH, Ahn S, Park DJ, Kim H, Kim SH (2016). Prognostic implications of immunosuppressive protein expression in tumors as well as immune cell infiltration within the tumor microenvironment in gastric cancer. Gastric Cancer.

[10] Zhou Y, Zhu G, Lu X, Zheng KI, Wang Q, Chen J (2020). Identification and validation of tumour microenvironment-based immune molecular subgroups for gastric cancer: immunotherapeutic implications. Cancer Immunol Immunother.

[11] Kelly RJ (2017) Immunotherapy for esophageal and gastric cancer. In: Dizon DS (ed) American society of clinical oncology educational book, vol 37. pp 292–300 10.1200/EDBK_175231 28561677

[12] Yu Y, Ma X, Zhang Y, Zhang Y, Ying J, Zhang W (2019). Changes in expression of multiple checkpoint molecules and infiltration of tumor immune cells after neoadjuvant chemotherapy in gastric cancer. J Cancer.

[13] Li Z, Gao X, Peng X, Chen MM, Zhe Li, Wei B (2020). Multi-omics characterization of molecular features of gastric cancer correlated with response to neoadjuvant chemotherapy. Sci Adv.

[14] Ocana A, Nieto-Jiménez C, Pandiella A, Templeton AJ (2017). Neutrophils in cancer: prognostic role and therapeutic strategies. Mol Cancer.

[15] Mellor KL, Powell AGMT, Lewis WG (2018). Systematic review and meta-analysis of the prognostic significance of neutrophil-lymphocyte ratio (NLR) after r0 gastrectomy for cancer. J Gastrointest Cancer.

[16] Fukuda K, Tsujitani S, Maeta Y, Yamaguchi K, Ikeguchi M, Kaibara N (2002). The expression of RCAS1 and tumor infiltrating lymphocytes in patients with T3 gastric carcinoma. Gastric Cancer.

[17] Li F, Sun Y, Huang J, Xu W, Liu J, Yuan Z (2019). CD4/CD8 + T cells, DC subsets, Foxp3, and IDO expression are predictive indictors of gastric cancer prognosis. Cancer Med.

[18] Hashimoto T, Kurokawa Y, Takahashi T, Miyazaki Y, Tanaka K, Makino T (2019). Predictive value of MLH1 and PD-L1 expression for prognosis and response to preoperative chemotherapy in gastric cancer. Gastric Cancer.

[19] Wang Y, Zhu C, Song W, Li J, Zhao G, Cao H (2018). PD-L1 expression and CD8+ T Cell infiltration predict a favorable prognosis in advanced gastric cancer. J Immunol Res.

[20] Strippoli A, Cocomazzi A, Basso M, Cenci T, Ricci R, Pierconti F (2020). c-MYC expression is a possible keystone in the colorectal cancer resistance to EGFR inhibitors. Cancers.

[21] Rossi ED, Martini M, Capodimonti S, Cenci T, Straccia P, Angrisani B (2014). Analysis of immunocytochemical and molecular BRAF expression in thyroid carcinomas: a cytohistologic institutional experience. Cancer Cytopathol.

[22] Martini M, Cenci T, D'Alessandris GQ, Cesarini V, Cocomazzi A, Ricci-Vitiani L (2013). Epigenetic silencing of Id4 identifies a glioblastoma subgroup with a better prognosis as a consequence of an inhibition of angiogenesis. Cancer.

[23] Zhu Y, Sun Y, Hu S, Jiang Y, Yue J, Xue X (2017). Comparison of five tumor regression grading systems for gastric adenocarcinoma after neoadjuvant chemotherapy: a retrospective study of 192 cases from National Cancer Center in China. BMC Gastroenterol.

[24] Noble F, Lloyd MA, Turkington R, Griffiths E, O'Donovan M, O'Neill JR (2017). Multicentre cohort study to define and validate pathological assessment of response to neoadjuvant therapy in oesophagogastric adenocarcinoma. Br J Surg.

[25] Corbeau I, Jacot W, Guiu S (2020). Neutrophil to lymphocyte ratio as prognostic and predictive factor in breast cancer patients: a systematic review. Cancers.

[26] Nejati R, Goldstein JB, Halperin DM, Wang H, Hejazi N, Rashid A (2017). Prognostic significance of tumor-infiltrating lymphocytes in patients with pancreatic ductal adenocarcinoma treated with neoadjuvant chemotherapy. Pancreas.

[27] Li Y, Wei Y, He Q, Wang X, Fan C, Li G (2018). Clinicopathological and prognostic significance of high circulating lymphocyte ratio in patients receiving neoadjuvant chemotherapy for advanced gastric cancer. Sci Rep.

[28] Taube JM, Anders RA, Young GD, Xu H, Sharma R, McMiller TL (2012). Colocalization of inflammatory response with B7–h1 expression in human melanocytic lesions supports an adaptive resistance mechanism of immune escape. Sci Trans Med.

[29] Thompson ED, Zahurak M, Murphy A, Cornish T, Cuka N, Abdelfatah E (2017). Patterns of PD-L1 expression and CD8 T cell infiltration in gastric adenocarcinomas and associated immune stroma. Gut.

[30] Galluzzi L, Buqué A, Kepp O, Zitvogel L, Kroemer G (2015). Immunological effects of conventional chemotherapy and targeted anticancer agents. Cancer Cell.

[31] Saito R, Abe H, Kunita A, Yamashita H, Seto Y, Fukayama M (2017). Overexpression and gene amplification of PD-L1 in cancer cells and PD-L1+ immune cells in Epstein–Barr virus-associated gastric cancer: the prognostic implications. Mod Pathol.

[32] Gu L, Chen M, Guo D, Zhu H, Zhang W, Pan J (2017). PD-L1 and gastric cancer prognosis: a systematic review and meta-analysis. PLoS ONE.

[33] Al-Batran SE, Homann N, Pauligk C, Goetze TO, Meiler J, Kasper S (2019). Perioperative chemotherapy with fluorouracil plus leucovorin, oxaliplatin, and docetaxel versus fluorouracil or capecitabine plus cisplatin and epirubicin for locally advanced, resectable gastric or gastro-oesophageal junction adenocarcinoma (FLOT4): a randomised, phase 2/3 trial. Lancet.

[34] Wang L, Zhang Q, Ni S, Tan C, Cai X, Huang D (2018). Programmed death-ligand 1 expression in gastric cancer: correlation with mismatch repair deficiency and Her2-negative status. Cancer Med.

[35] Wang F, Liu Z, Xia Y, Zhou C, Shen X, Li X (2015). Changes in neutrophil/lymphocyte and platelet/lymphocyte ratios after chemotherapy correlate with chemotherapy response and prediction of prognosis in patients with unresectable gastric cancer. Oncol Lett.

[36] Li Z, Li S, Ying S, Zhang L, Shan F, Jia Y (2020). The clinical value and usage of inflammatory and nutritional markers in survival prediction for gastric cancer patients with neoadjuvant chemotherapy and D2 lymphadenectomy. Gastric Cancer.

[37] Elfving H, Mattsson JSM, Lindskog C, Backman M, Menzel U, Micke P (2019). Programmed cell death ligand 1 immunohistochemistry: a concordance study between surgical specimen, biopsy, and tissue microarray. Clin Lung Cancer.

[38] Ye M, Huang D, Zhang Q, Weng W, Tan T, Qin G (2020). Heterogeneous programmed death-ligand 1 expression in gastric cancer: comparison of tissue microarrays and whole sections. Cancer Cell Int.

[39] Lee JS, Won HS, Sun DS, Hong JH, Ko YH (2018). Prognostic role of tumor-infiltrating lymphocytes in gastric cancer: a systematic review and meta-analysis. Medicine (Baltimore).

[40] Chakrabarti J, Holokai L, Syu L, Steele N, Chang J, Dlugosz A (2018). Mouse-derived gastric organoid and immune cell co-culture for the study of the tumor microenvironment. Methods Mol Biol.

