# Supplementary material for: Predictive value of NLR, TILs (CD4+/CD8+) and PD-L1 expression for prognosis and response to preoperative chemotherapy in gastric cancer
Source: Cancer Immunol Immunother. 2021 May 19;71(1):45–55. doi: 10.1007/s00262-021-02960-1 (PMC8738448; doi:10.1007/s00262-021-02960-1)
Supplement: Supplementary file 1 — Supplementary file1 (DOCX 21 kb) [file 262_2021_2960_MOESM1_ESM.docx]

**SUPPLEMENTARY MATERIAL**

**Patients’ features**

Inclusion criteria were: 1) diagnosis of locally advanced GC; 2) histologically confirmed adenocarcinoma; 3) treatment with NAD-CT; 4) known histotype, according to Lauren’s classification (intestinal *vs* diffuse); 5) availability of baseline (i.e. performed at diagnosis, before NAD administration) complete blood count; 6) availability of baseline multisite sampling tumor tissue biopsies (containing at least 2 pre-treatment endoscopic biopsies, median 4.02, with at least 50% tumor tissue); 7) availability of pretreatment CT. Patients with altered expression of mismatch repair proteins (MMRP, including PMS2, hMLH1, hMSH6 and hMSH2), EBV positive GC, and whose histotype according to Lauren‘s classification was unknown, and with evidence of metastatic disease or history of other cancers (with the exception of non-melanoma skin cancers and *in situ* cervical cancer) diagnosed within the previous 5 years, were excluded. The treatment was decided after multidisciplinary team (MDT) evaluation for each patient*.* Patients with gastric cancer stage II received NAD-CT were not included in our cohort because of the limited number of cases (only two patients with all inclusion criteria).

Twenty-one patients underwent total gastrectomy (32.3%) and the remaining 44 patients underwent sub-total gastrectomy (67.7%). According to the Clavien-Dindo Classification grade, ten patients (15.4%) had low or mild postoperative complications (Grade I-II), while seven patients (10.7%) had major postoperative complications (Grade I-II)*.

All patients received NAD-CT (ACCORD protocol with few modifications) consisting of three preoperative cycles of fluorouracil 800 mg/m^2^ as continuous intravenous (IV) infusion for 5 consecutive days (days 1 to 5) and cisplatin 75 mg/m^2^ every 21 days followed by 3 postoperative cycles in case of good tolerance and no evidence of progressive disease after preoperative chemotherapy, for a total of 6 cycles (CF-regiment). All patients completed two cycles of NAD-CT. There were no treatment-related deaths. The most frequent toxicities were nausea, vomiting, and diarrhea. About the hematological adverse events, 14% of patients experienced grade 3–4 anemia and leukopenia. No abnormal results for liver or renal function tests were observed in grade 3–4 anemia. All patients underwent surgery and 56 patients (86%) achieved R0 resection.

Post-treatment follow-up examinations were performed according to the institutional protocol, and tumor markers carcinoembryonic antigen (CEA) and CA19-9 were examined every 3 months. Chest X-ray and abdominal/pelvic CT scan were performed every 6 months. Gastroscopy was also required each year. Positron emission tomography (PET) scan was carried out when recurrence was suspected. All patients were followed up every 3 months in the first 2 years, 6 months in the 3-5 years, and 12 months annually thereafter, the median follow-up time was 26.7 months (range, 2.5-131).

All patient data were collected anonymously; the study was conducted in accordance with the Declaration of Helsinki. Before the first chemotherapy administration, all patients signed an informed consent approved by the Ethics Committee of “Università Cattolica del Sacro Cuore-Roma”. In this study we follow the REMARK criteria to individuate a new biomarker**.

**HER2 gene amplification**

The DISH assay was performed according to the manufacturer's recommended protocol for surgical specimens. The HER2/neu (black) to chromosome enumeration probe 17 (CEP17) (red) ratio was manually counted using a light microscope and the result was confirmed by a second investigator. At least 20 cells were counted. The criteria consist of a combination of the HER2/CEP17 ratio and the average number of HER2 signals per cell. The HER2 gene amplification was scored as “amplified” if the case had a HER2/CEP17 signal count ratio of 2.0 or if the HER2/CEP17 signal count ratio was <2.0 but the average number of HER2 signals per cell was 6.0. A score of “equivocal” was given if the case had a HER2/CEP17 signal count ratio of <2.0 and the average number of HER2 signals per cell was ≥4.0 and <6.0. A score of “not amplified” was given if the case had a HER2/CEP17 signal count ratio of <2.0 and the average number of HER2 signals was <4.0 [20].

**TILs (CD4+/CD8+ T-cells ratio) evaluation and PD-L1 expression**

Briefly, 4-μm sections were obtained from FFPE blocks and mounted on positive charged glass slides. For antigen retrieval, deparaffinized and rehydrated sections were treated with citric acid buffer (pH 6.0), 2 cycles of 5 minutes each at 750 W, followed by inhibition of endogenous peroxidase with 3% H_2_O_2_ for 5 minutes. Then, the sections were incubated for 20 minutes at room temperature with a prediluted mouse anti-CD4 monoclonal antibody (Clone 4B12; Dako, Carpinteria, CA) or a prediluted mouse anti-CD8 monoclonal antibody (Clone 1A5; Ventana Inc. Tucson, AZ). The primary antibodies were visualized using the avidin–biotin–peroxidase complex method (UltraTek HRP Anti-polyvalent; ScyTek, Logan, Utah) according to the instruction manual. 3,3’-Diaminobenzidine was used as the enzyme substrate to observe the specific antibody localization and Mayer hematoxylin was used as a nuclear counterstain. Human lymph-node or tonsil tissues were used as a positive control, whereas the negative control was prepared by replacing the primary antibody with a nonimmune immunoglobulin of the same isotype. Quantification of CD4+ and CD8+ T cells was performed microscopically and expressed as the average value on 10 HPFs for each lineage. The results of IHC reactions were independently evaluated by two pathologists (MS and MM) that were blinded to clinicopathologic data. Variations in the enumeration, within a range of 5%, were re-evaluated and a consensus decision was made. CD4+/CD8+ T-cell tissue ratio was calculated dividing CD4+ T-cell mean value by CD8+ T-cell one, for each specimen. The immunohistochemically stained samples were examined under a light microscope (Olympus BX-51). For each immunohistochemically stained tissue, the tumor infiltrating lymphocytes were counted in 10 different areas under 400× magnification. Only TILs within the borders of the invasive tumor were evaluated (within 0.5 mm of the tumor). The assessment focused on tumor areas and ulcers, ulcer floor areas, necrotic area and high-grade epithelial dysplasia/carcinoma in situ were excluded from the evaluation. Since immunoreactivity was observed also in the tumor epithelium during the evaluation of CD4+ cells, only those displaying lymphocyte morphology were considered. Immune cells’ count was collected for each of the 10 fields and then averaged to calculate the mean number for 1 computerized 400× microscopic field (0.1590 mm2/field).

PD-L1 expression was evaluated using immunohistochemistry (IHC) and anti-PD-L1 (22C3) rabbit monoclonal antibody (PD-L1 IHC 22C3 pharmDX; Agilent Technolologies, Carpinteria, CA, USA). Formalin-fixed, paraffin-embedded blocks (FFPE) were cut into 4-mm sections. PD-L1 IHC was performed with Dako’s autostainer link48 according to the manufacturer’s instructions. PD-L1 expression was evaluated on tumor and immune cells according to the CPS score. CPS is the sum of PD-L1–stained cells’ number (tumor cells, lymphocytes, macrophages) divided by the total number of viable tumor cells, multiplied by 100, as in the formula below:

CPS= number of PD-L1 stained cells (tumor cells, lymphocytes, macrophages) x 100

                          Total number of viable tumor cells

At least 100 viable tumor cells must be present in the PD-L1–stained slide for the specimen to be considered adequate for evaluation. Tumor cells must show partial or complete membrane staining to be counted as ‘‘stained,’’ whereas immune cells are counted if any staining appeared. Moreover, complying with CPS exclusion criteria, we specifically ruled out any PD-L1-positive immune cell associated either with adenoma, dysplasia, with ulcers, chronic gastritis, or any other process not directly attributable to the tumor.

The agreement indices (Cohen’s K) between the two pathologists (M.M. and R.R., who also went through a formal training program to evaluate CPS by the 22C3 pharmDx assay) who evaluated the TILs and PD-L1 expression, were very good: k=0.82 and k=0.87, respectively.

**Statistical Analysis**

Statistical comparison of continuous variables was performed by the Mann-Whitney U-test (t test), as appropriate. Comparison of categorical variables was performed by chi-square statistic, using the Fisher’s exact test. To evaluate the agreement between the two pathologists about the TILs ratio and PD-L1 score, the inter-rater agreement (Kappa) was calculated using MedCalc software. Pearson correlation coefficient (r) was employed to evaluate the association between two or more variables.

Kaplan-Meier survival curves were plotted and differences in survival between groups of patients were compared using the log-rank test. Multivariate analysis was performed using the Cox proportional hazards regression analysis including only those clinical and biological variables with a p-value of 0.10 or lower at the univariate analysis. P-values less than 0.05 were considered as statistically significant.

* Dindo D, MD, Demartines N, Clavien P. Classification of Surgical Complications: A New Proposal With Evaluation in a Cohort of 6336 Patients and Results of a Survey. Ann Surg. 2004;240:205–213.

** Sauerbrei W, Taube SE, McShane LM, Cavenagh MM, Altman DG. Reporting Recommendations for Tumor Marker Prognostic Studies (REMARK): An Abridged Explanation and Elaboration. J Natl Cancer Inst. 2018;110:803-811.

**Supplementary figure 1 legend**

Panel A: the figure shows the changes in NRL level between pre- and post-treatment LAGC (p=0.0033, paired t-test); Panel B and C: Kaplan-Meier curves for PFS (panel B) and OS (panel C) of NAD-CT treated LAGC patients stratified by post-treatment NLR. Patients with lower post-NLR<2.5, (blue-line) was significantly associated to a better PFS (p=0.001) and OS (p=0.015) respect to those patients with post-treatment NLR≥2.5 (red-line).

**Supplementary figure 2 legend**

Panel A: the figure shows the direct and significant correlation between NLR and CD4+/CD8+ T cells tissue ratio (TILs) (Spearman r= 0.6338; p<0.0001); Panel B: the figure shows the indirect and significant correlation between NLR and PD-L1 level (Spearman r= -0.781; p<0.0001); Panel C: the figure shows the indirect and significant correlation between TILs and PD-L1 level (Spearman r= -0.567; p<0.0001).
