# Supplementary figures and images for: Predictive value of NLR, TILs (CD4+/CD8+) and PD-L1 expression for prognosis and response to preoperative chemotherapy in gastric cancer
Source: Cancer Immunol Immunother. 2021 May 19;71(1):45–55. doi: 10.1007/s00262-021-02960-1 (PMC8738448; doi:10.1007/s00262-021-02960-1)

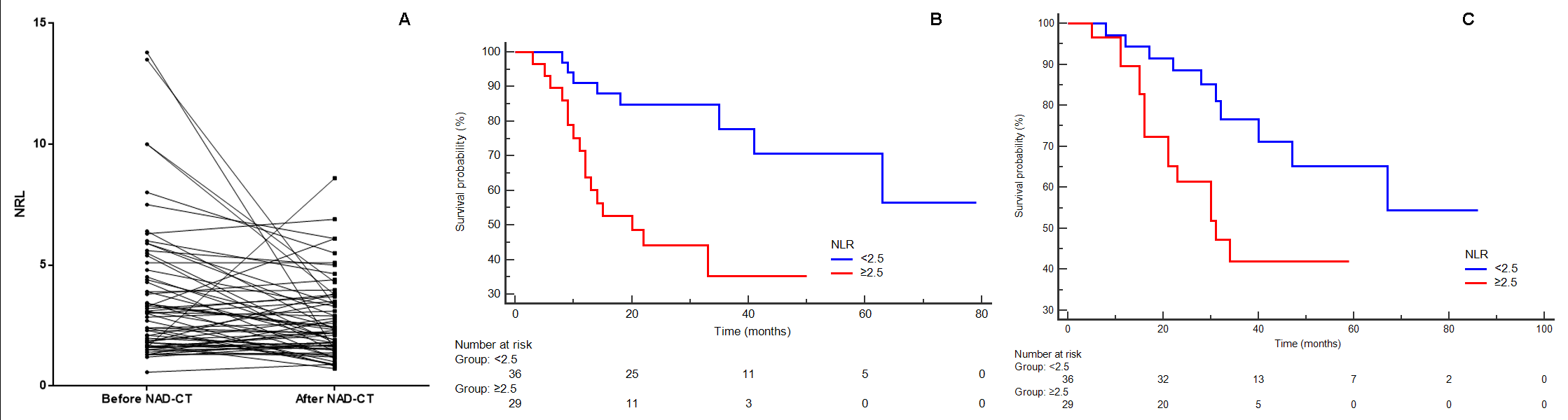

Supplement: Supplementary file 2 — Supplementary file2 (TIF 4319 kb) [file 262_2021_2960_MOESM2_ESM.tif]

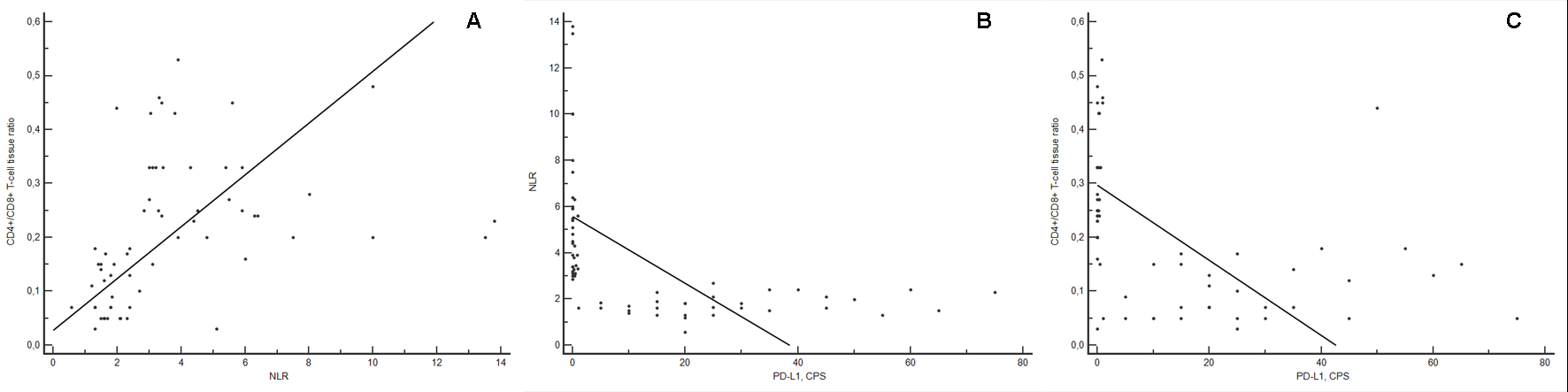

Supplement: Supplementary file 3 — Supplementary file3 (TIF 2523 kb) [file 262_2021_2960_MOESM3_ESM.tif]
