# Supplementary material for: Predictive value of NLR, TILs (CD4+/CD8+) and PD-L1 expression for prognosis and response to preoperative chemotherapy in gastric cancer
Source: Cancer Immunol Immunother. 2021 May 19;71(1):45–55. doi: 10.1007/s00262-021-02960-1 (PMC8738448; doi:10.1007/s00262-021-02960-1)
Supplement: Supplementary file 5 — Supplementary file5 (DOCX 18 kb) [file 262_2021_2960_MOESM5_ESM.docx]

|  |  | **PFS** |  | |  | **OS** |  |
| --- | --- | --- | --- | --- | --- | --- | --- |
|  |  |  | |  |  |  |  |
|  | **HR** | **95% CI** | | **P** | **HR** | **95% CI** | **P** |
| **Age** |  |  | |  |  |  |  |
| ≥ 63 vs < 63 | 1.1083 | 0.4853 to 2.5313 | | 0.8072 | 0.8070 | 0.3616 to 1.8012 | 0.6007 |
| **Gender** |  |  | |  |  |  |  |
| Male vs Female | 2.1326 | 0.9283 to 4.8989 | | 0.0743 | 1.3521 | 0.6012 to 3.0407 | 0.4657 |
| **TNM stage** |  |  | |  |  |  |  |
| IIIB vs IIIC | 0.4208 | 0.1859 to 0.9526 | | **0.0379** | 0.3760 | 0.1679 to 0.8417 | **0.0174** |
| **Tumor site** |  |  | |  |  |  |  |
| Upper vs Middle-Lower | 1.0966 | 0.4812 to 2.4990 | | 0.8264 | 1.3976 | 0.6229 to 3.1358 | 0.4169 |
| **Histotype** |  |  | |  |  |  |  |
| Intestinal vs Diffuse | 0.6317 | 0.2756 to 1.4479 | | 0.2777 | 0.4849 | 0.2167 to 1.0852 | 0.0782 |
| **HER2 status** |  |  | |  |  |  |  |
| HER2 + vs HER2 - | 0.8989 | 0.3438 to 2.3505 | | 0.8280 | 0.7530 | 0.3031 to 1.8709 | 0.5412 |
| **LVI** |  |  | |  |  |  |  |
| Yes vs No | 2.2800 | 0.9948 to 5.2257 | | **0.0515** | 2.6129 | 1.1661 to 5.8550 | **0.0196** |
| **PNI** |  |  | |  |  |  |  |
| Yes vs No | 1.7855 | 0.7141 to 4.4645 | | 0.2151 | 1.4812 | 0.6149 to 3.5680 | 0.3811 |
| **TRG** |  |  | |  |  |  |  |
| TRG 1-2 vs TRG 3-5 | 2.7022 | 1.1373 to 6.4201 | | **0.0244** | 2.9778 | 1.2817 to 6.9182 | **0.0112** |

**Table 2S.** Correlation between clinical and biological parameters and PFS (months) and OS (months).

Abbreviations: LVI. linfovascular invasion; PNI. perineural infiltration.
